# A141 TREATMENT OPTIONS FOR LYMPHOCYTIC ESOPHAGITIS: A SYSTEMATIC REVIEW OF CASE REPORTS AND CASE SERIES

**DOI:** 10.1093/jcag/gwae059.141

**Published:** 2025-02-10

**Authors:** B A Nguyen, F Jowhari

**Affiliations:** The University of British Columbia Faculty of Medicine, Vancouver, BC, Canada; Medicine, The University of British Columbia Okanagan, Kelowna, BC, Canada

## Abstract

**Background:**

Lymphocytic esophagitis (LyE) is a novel rare disorder characterized by intraepithelial lymphocytic infiltration of the esophagus in a peripapillary distribution without granulocytes, first described by Rubio et al. in 2006. No systematic reviews or randomized controlled trials have been published to date on the treatment options for LyE.

**Aims:**

To describe the current state of evidence published to date for the treatment of lymphocytic esophagitis.

**Methods:**

We performed a systematic review according to PRISMA guidelines, searching MEDLINE, Embase, Scopus, and Web of Science. Descriptive statistics were performed after extracting data on the study design, patient characteristics, treatment modalities, and outcomes (symptomatic, endoscopic, and histologic). The absence of randomized controlled trials and heterogeneity in outcome measures precluded the ability to perform a formal meta-analysis.

**Results:**

A total of 39 articles (36 case reports, 3 case series) from 2012-2024 were selected for analysis. Proton pump inhibitors (PPIs) were the most common initial therapy used in 73.2% of patients (60/82), followed by topical steroids in 26.8% (22/82). A greater proportion of patients experienced a symptomatic, histologic and endoscopic response from initial use of topical steroids as monotherapy or part of PPI/Steroids combination therapy compared to PPIs alone. Histologic response was highest in the steroid monotherapy group (**Table 1**). Symptomatic recurrence was more common after initial use of topical steroids (as monotherapy or part of combination therapy) compared to PPIs alone (**Table 1**) but the samples sizes were very small. Initial endoscopic dilation improved symptoms in 90% (9/10) of patients and 66.7% (4/6) had successful stricture resolution, but 25% of patients experienced symptomatic recurrence. Other therapies for LyE included Vedolizumab, Botox injection, Sucralfate and Tacrolimus. The average follow-up duration was 8.98 months.

**Conclusions:**

For patients with histologically confirmed LyE, both PPIs and topical steroids are viable options for symptomatic relief, with topical steroids showing higher symptomatic and histologic improvement. Balloon dilation may alleviate dysphagia in cases of obvious strictures. Given the limited available data, further research, including randomized controlled trials, is necessary to better define optimal treatment targets (histologic vs. symptomatic outcomes) and to formally compare treatment efficacies.

Table 1: Initial treatment response and symptomatic recurrence

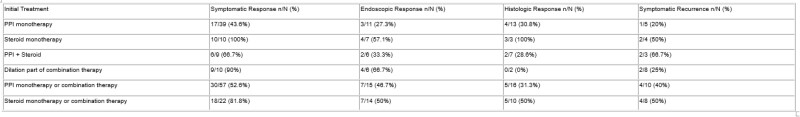

**Funding Agencies:**

None

